# Fine mapping of genetic polymorphisms of pulmonary tuberculosis within chromosome 18q11.2 in the Chinese population: a case-control study

**DOI:** 10.1186/1471-2334-11-282

**Published:** 2011-10-22

**Authors:** Yaoyao Dai, Xia Zhang, Hongqiu Pan, Shaowen Tang, Hongbing Shen, Jianming Wang

**Affiliations:** 1 Department of Epidemiology and Biostatistics, School of Public Health, Nanjing Medical University, Nanjing, 210029 PR China; 2 Department of Tuberculosis, Nanjing Chest Hospital, Nanjing, 210029 PR China; 3 Department of Tuberculosis, Third Hospital of Zhenjiang City, Zhenjiang, 212005 PR China

## Abstract

**Background:**

Recently, one genome-wide association study identified a susceptibility locus of rs4331426 on chromosome 18q11.2 for tuberculosis in the African population. To validate the significance of this susceptibility locus in other areas, we conducted a case-control study in the Chinese population.

**Methods:**

The present study consisted of 578 cases and 756 controls. The SNP rs4331426 and other six tag SNPs in the 100 Kbp up and down stream of rs4331426 on chromosome 18q11.2 were genotyped by using the Taqman-based allelic discrimination system.

**Results:**

As compared with the findings from the African population, genetic variation of the SNP rs4331426 was rare among the Chinese. No significant differences were observed in genotypes or allele frequencies of the tag SNPs between cases and controls either before or after adjusting for age, sex, education, smoking, and drinking history. However, we observed strong linkage disequilibrium of SNPs. Constructed haplotypes within this block were linked the altered risks of tuberculosis. For example, in comparison with the common haplotype AA_(rs8087945-rs12456774)_, haplotypes AG_(rs8087945-rs12456774) _and GA_(rs8087945-rs12456774) _were associated with a decreased risk of tuberculosis, with the adjusted odds ratio(95% confidence interval) of 0.34(0.27-0.42) and 0.22(0.16-0.29), respectively.

**Conclusions:**

Susceptibility locus of rs4331426 discovered in the African population could not be validated in the Chinese population. None of genetic polymorphisms we genotyped were related to tuberculosis in the single-point analysis. However, haplotypes on chromosome 18q11.2 might contribute to an individual's susceptibility. More work is necessary to identify the true causative variants of tuberculosis.

## Background

After two decades of neglect, tuberculosis (TB) is being resurrected as a major public health problem, especially in low- and middle-income countries [1]. Nearly one third of the world's population has been latently infected with the pathogen of mycobacterium tuberculosis (MTB) [2]. However, only 10% of them will develop active TB throughout their lifetimes [3-5]. Although previous studies have indicated that susceptibility has a substantial genetic component [6-8], progress in the determination of contributing genetic variants of TB was slow. With the completion of Human Genome Project and advances in genotyping technology, Genome-wide Association (GWA) Study has been one powerful tool for the study of genetic susceptibility in human complex diseases [9]. Despite the widely held view that exposure to pathogens during human evolution has put evolutionary pressures on host susceptibility, progress in identifying susceptibility genes for infectious diseases has been slow in comparison to other common disorders [10]. Recently, a GWA study in Ghana and Gambia identified a susceptibility locus of rs4331426 on chromosome 18q11.2 in association with the risk of TB [odds ratio (OR) = 1.19, 95% confidence interval (CI):1.13-1.27, *P *= 6.8 × 10^-9^] [11]. However, till now this finding has not been replicated in other populations.

China, the world's second largest country with TB epidemic, has a different genetic background, lifestyle, and disease prevalence from Africa [12]. It was estimated that 1.3 million people across the country developed active TB in 2009, of whom 600 000 had the highly infectious form. To validate the findings from the GWA study in Ghana and Gambia and to search for more susceptibility loci of TB, we performed a case-control study via the fine mapping analysis of the region of 18q11.2 in the Chinese population.

## Methods

### Study population

This case-control study was conducted in Jiangsu, a developed province located in the eastern part of China, with a total population of 77 million in 2009. We recruited 578 patients with pulmonary TB, including 368 (63.7%) new cases and 210 (36.3%) previously treated ones. For the definitions of new and previously treated cases, we referred to the WHO guidelines. In brief, a "new case" was termed as a newly registered episode of TB in a patient who, in response to direct questioning, denied having had any prior antituberculosis treatment (for up to one month); and in study sites where adequate documentation was available, there was no evidence of such history. A "previously treated case" was defined as a newly registered episode of TB in a patient who, in response to direct questioning admitted having been treated for TB for one month or more, or, in study sites where adequate documentation was available, there was evidence of such history. All patients were diagnosed with the evidence of sputum culture. Sputum samples were cultured on Lowenstein-Jensen (LJ) culture media. Identification of MTB was done by using the p-nitrobenzoic acid (PNB) and thiophene carboxylic acid hydrazine (TCH) resistance test. Growth in LJ medium containing PNB indicated that the bacilli did not belong to the MTB complex. We also recruited 756 controls from a pool of individuals who participated in the local community-based health examination program. Controls were frequency-matched to the cases by sex and age. These control subjects had no self-reported history of TB, diabetes and malignancy. All cases and controls had no prior HIV positive history. Each subject was individually interviewed in local health facilities by using a structured questionnaire and donated a blood sample for genotyping analysis.

### SNPs selection and genotyping

The significant SNP rs4331426, which was identified in a GWA study in Ghana and Gambia, is located on the region of chromosome 18q11.2 [11]. Due to the low minor allele frequency (MAF) of this SNP (< 5%) among Han Chinese, we further searched for tag SNPs around it. Firstly, we downloaded all eligible SNPs in the 100 Kbp up and down stream of the SNP rs4331426 on chromosome 18q11.2 by using the Chinese Han population (CHB) database of HapMap http://www.hapmap.org/. All SNPs in this region were filtered by using the following criteria: (1) MAF≥0.05; (2) Hardy-Weinberg equilibrium test *P *value≥0.05. Then, tag SNPs were selected by using Haploview 4.2 software based on their ability to tag surrounding variants [13]. As a result, 7 SNPs (rs4330012, rs8087945, rs12456774, rs12457731, rs12958098, rs4800136 and rs4800417) were chosen for genotyping, which was performed by using TaqMan allelic discrimination technology on the ABI 7900 Real-Time PCR System (Applied Biosystems, Foster City, CA) [14]. The primers and probes for each SNP (Table 1) were designed by Nanjing Steed BioTechnologies Co., Ltd. Due to the technical limitation, we failed to design probes for detecting the SNP rs4330012 as another SNP rs9954441 neighboring to it. Preliminary experiments were carried out for each SNP and blank controls were set in each batch of samples. Both the laboratory personnel and the readers of genotyping were blinded to the status of cases and controls. The overall call rate of genotyping was > 95%.

**Table 1:** Information of primers and probes

SNPs	Primer sequence(5'-3')	Probe sequence
rs8087945	F-GGATGACAGAGTGACTGACACACA	C: FAM-AGAAAGAATCTTGCACTTT-MGB
A > G	R-AGAAGCAGCAAGAAGTGGGTG	T: HEX-AGAAAGAATTTTGCACTTTA-MGB
rs12456774	F-AGCTTTGATACCCCAGCATAGG	C: FAM-CAACTTAATTCGCGTCTT-MGB
A > G	R-TTGGCAATGAATTTTCCATGAG	T: HEX-CCAACTTAATTCGTGTCTT-MGB
rs12457731	F-ACCATCTTAATGTCTTCGATTGAAGT	G: FAM-CCTTATCCTAGATTCTTAGC-MGB
A > G	R-TAGATGCTTTCCTCCAGGAGCTA	A: HEX-CCTTATCCTAAATTCTTAGC-MGB
rs12958098	F-TCTGCAAGAGGCTTGAGGCTAT	A: FAM-CAATTCCCAGCAGTCT-MGB
C > T	R-GTAAACCAAAGCTCACAGTTTCAATT	G: HEX-CAATTCCCGGCAGTC-MGB
rs4800136	F-CACCTGACCAACAAAAGGAGTG	G: FAM-CTCTTTGTTGTTAATACCT-MGB
A > C	R-GACTAGAAGCACAAAAGCCATCTC	T: HEX-CTCTTTGTTTTTAATACCT-MGB
rs4800417	F-GTGCCAGCTCTGTGGTTAGCT	G: FAM-TCAACCTTGCCAAGC-MGB
C > T	R-TCCCCACTCTTACTCATTTGAAAGA	A: HEX-TCCACTCAACCTTACCA-MGB
rs4331426	F-GGTTTGGATTAACAGGCAAGAAA	A: FAM-TACCAATGCACAGGAT-MGB
A > G	R-ACCACCTCTTGTAGATGAGAAAATTAAA	G: HEX-TACCAATGCGCAGGAT-MGB

### Statistical analysis

Data were double entered with EpiData 3.1 (Denmark) and discrepancies were checked against the raw data. Continuous variables were described as mean ± SD and differences between groups were analyzed by using student-*t *test. Categorized variables were described as percentage and analyzed by using the Chi-square test. Unconditional logistic regression model was used to calculate odds ratio (OR) and 95% confidence interval (CI), as well as corresponding *P*-values. Hardy-Weinberg equilibrium was estimated using the χ^2 ^goodness of fit test among controls. Haplotype blocks were selected with Haploview software by considering linkage disequilibrium (LD) blocks. The estimated frequency of polymorphic loci was calculated using PHASE 2.1 software. All analyses were performed using the SPSS software (SPSS Inc., USA). The *P*-value reported was two-sided and the values less than 0.05 were considered statistically significant.

### Ethical consideration

This project has been approved by the Institutional Review Board of Nanjing Medical University. Written informed consents were obtained from all participants. Ethics has been respected throughout the whole study period.

## Results

Overall, this study consisted of 578 cases (72.1% males, 27.9% females) and 756 controls (75.3% male, 24.7% female). The age (mean ± SD) was 52.07 ± 18.01 years for cases and 52.85 ± 18.42 years for controls, respectively. As a result of frequency-matching, there were no significant differences in the distribution of gender and age between cases and controls. However, education level and the history of smoking and drinking were found to be different between the two groups. As shown in Table 2, the proportion of ever smoking was 52.5% in patients, which was significantly higher than that in controls (44.4%) (χ^2 ^= 8.543, *P *= 0.003). In contrast, the proportion of alcohol drinking was 17.9% in TB patients, which was significantly lower than that in controls (28.4%) (χ^2 ^= 19.675, *P *< 0.001).

**Table 2:** Basic characteristics of the cases and controls

	case(N = 578)		control(N = 756)		
		
Variables	**No**.	%	**No**.	%	*P*
**Age **(mean ± SD)	52.07 ± 18.01		52.85 ± 18.42		0.176
**Gender**					0.199
Male	417	72.1	569	75.3	
Female	161	27.9	187	24.7	
**Education**					**< 0.001**
Illiterate or semi-illiterate	164	28.4	89	11.8	
Primary school	133	23	215	28.4	
Junior high school	201	34.8	267	35.3	
Senior high school or above	80	13.8	185	24.5	
**Smoking**					**0.003**
Ever	299	52.5	336	44.4	
Never	270	47.5	420	55.6	
**Drinking**					**< 0.001**
Ever	101	17.9	215	28.4	
Never	463	82.1	541	71.6	

As expected, genetic variants of rs4331426 were rare in the study population. The frequencies of AA, AG, and GG genotype were 93.70%, 6.14%, and 0.15%, respectively. No significant difference was observed in the distribution of either genotypes or alleles of this SNP between cases and controls. Six tag SNPs we genotyped were all in Hardy-Weinberg equilibrium [rs8087945 (χ^2 ^= 0.330, *P *= 0.566), rs12456774 (χ^2 ^= 0.438, *P *= 0.508), rs12457731 (χ^2 ^= 0.236, *P *= 0.627), rs12958098 (χ^2 ^= 0.757, *P *= 0.384), rs4800136 (χ^2 ^= 0.047, *P *= 0.829) and rs4800417 (χ^2 ^= 1.320, *P *= 0.251)]. The genotype analysis showed that the minor allele frequencies of rs8087945, rs12456774, rs12457731, rs12958098, rs4800136 and rs4800417 were 22.41%, 29.96%, 5.10%, 47.14%, 3.91% and 19.34% in the cases and 22.00%, 29.93%, 4.37%, 48.99%, 4.03% and 18.66% in the controls, respectively. No significant difference was observed in genotypes or allele frequencies of the six tag SNPs between case and control groups either before or after adjusting for age, sex, education, smoking, and drinking history (Table 3). Side by side r^2^/D' plot for six tag SNPs was shown in the additional file 1. By considering both D' and r^2^, we analyzed two haplotypes as presented in the Table 4. For example, strong LD was observed between rs8087945 and rs12456774 (D' = 1, r^2 ^= 0.12). The common haplotype within this block was AA _(rs8087945-rs12456774)_. Comparison of haplotype frequencies between case and control groups demonstrated that both the haplotype AG_(rs8087945-rs12456774) _and GA_(rs8087945-rs12456774) _were associated with the decreased risk of TB, with the adjusted OR(95% CI) of 0.34(0.27-0.42) and 0.22(0.16-0.29), respectively (Table 4). A strong linkage disequilibrium was also observed between rs4800417 and rs8087945 (D' = 0.99, r^2 ^= 0.78). As compared with the common haplotype CA_(rs4800417-rs8087945)_, the haplotype TG_(rs4800417-rs8087945) _was associated with a decreased risk (aOR = 0.64, 95%CI: 0.50-0.81) whereas the haplotype CG_(rs4800417-rs8087945) _was related to an increased risk of TB (OR = 3.19, 95%CI: 2.26-4.51) (Table 4).

**Table 3:** Distribution of genotypes in cases and controls and their risks with pulmonary tuberculosis

	Case		Control						Model		
						
SNP	N	%	N	%	cOR (95%CI)	*P*	**aOR (95%CI)**^**a**^	***P ***^**a**^	**Model**^**b**^	OR(95%CI)	*P*
rs8087945 A > G											
AA	338	60.36	459	61.20	1		1		Dom	1.02(0.80-1.29)	0.885
AG	193	34.46	252	33.60	1.04(0.82-1.31)	0.742	1.04(0.81-1.33)	0.763	Rec	0.88(0.52-1.48)	0.619
GG	29	5.18	39	5.20	1.01(0.61-1.67)	0.970	0.89(0.52-1.51)	0.660	Add	0.99(0.82-1.21)	0.946
rs12456774 A > G											
AA	272	48.66	372	49.60	1		1		Dom	1.01(0.80-1.27)	0.942
AG	239	42.75	307	40.93	1.06(0.85-1.34)	0.594	1.05(0.83-1.34)	0.675	Rec	0.81(0.54-1.21)	0.299
GG	48	8.59	71	9.47	0.92(0.62-1.38)	0.670	0.83(0.55-1.26)	0.376	Add	0.96(0.81-1.15)	0.683
rs12457731 A > G											
AA	524	90.66	691	91.52	1		1		Dom	1.13(0.76-1.69)	0.544
AG	49	8.48	62	8.21	1.04(0.70-1.54)	0.836	1.05(0.70-1.59)	0.811	Rec	3.42(0.65-18.02)	0.148
GG	5	0.86	2	0.27	3.30(0.64-17.05)	0.155	3.43(0.65-18.09)	0.146	Add	1.19(0.82-1.71)	0.36
rs12958098 C > T											
CC	162	28.98	199	26.82	1		1		Dom	0.87(0.68-1.13)	0.299
CT	267	47.76	359	48.38	0.91(0.70-1.19)	0.497	0.88(0.67-1.15)	0.341	Rec	0.94(0.72-1.24)	0.677
TT	130	23.26	184	24.80	0.87(0.64-1.18)	0.364	0.87(0.63-1.20)	0.387	Add	0.93(0.79-1.09)	0.370
rs4800136 A > C											
AA	520	92.36	696	92.06	1		1		Dom	0.94(0.61-1.44)	0.776
AC	42	7.46	59	7.81	0.95(0.63-1.44)	0.818	0.92(0.59-1.42)	0.702	Rec	2.39(0.15-39.23)	0.543
CC	1	0.18	1	0.13	1.34(0.08-21.45)	0.837	2.37(0.14-38.99)	0.546	Add	0.96(0.63-1.45)	0.838
rs4800417 C > T											
CC	366	65.24	503	66.80	1		1		Dom	1.09(0.85-1.38)	0.505
CT	173	30.84	219	29.08	1.09(0.85-1.38)	0.502	1.11(0.86-1.42)	0.434	Rec	0.91(0.50-1.66)	0.763
TT	22	3.92	31	4.12	0.98(0.56-1.71)	0.931	0.94(0.52-1.72)	0.844	Add	1.05(0.85-1.29)	0.646

a: Adjusted for age, sex, education, smoking and drinking

b: Dom, dominant model; Rec, recessive model; Add, additive mode

**Table 4:** Haplotype frequencies and the risks of pulmonary tuberculosis

Haplotypes	Case N (%)	Control N (%)	cOR (95% CI)	*P*	**aOR (95% CI)**^**a**^	***P***^**a**^
rs8087945- rs12456774						
A_rs8087945_A_rs12456774_	746(64.5)	725(47.9)	1		1	
A_rs8087945_G_rs12456774_	159(13.8)	454(30.0)	0.34(0.28-0.42)	**< 0.0001**	0.34(0.27-0.42)	**< 0.0001**
G_rs8087945_A_rs12456774_	75(6.5)	333(22.0)	0.22(0.17-0.29)	**< 0.0001**	0.22(0.16-0.29)	**< 0.0001**
G_rs8087945_G_rs12456774_	176(15.2)	0(0)	-	0.994	-	0.994

rs4800417-rs8087945						
C_rs4800417_A_rs8087945_	813(70.3)	1176(77.8)	1		1	
T_rs4800417_G_rs8087945_	126(10.9)	278(18.4)	0.66(0.52-0.82)	**0.0003**	0.64(0.50-0.81)	**0.0003**
C_rs4800417_G_rs8087945_	126(10.9)	55(3.6)	3.31(2.38-4.61)	**< 0.0001**	3.19(2.26-4.51)	**< 0.0001**
T_rs4800417_A_rs8087945_	91(7.9)	2(0.2)	43.88(13.84-139.07)	**< 0.0001**	48.08(15.02-153.92)	**< 0.0001**

a: Adjusted for age, sex, education, smoking and drinking.

## Discussion

A puzzling feature of TB is that only a small proportion of infected persons will develop active diseases during their lifetimes [15], though nearly one third of global populations have been latently infected with the pathogen [2]. Host genetic factors can explain, at least in part, why some people resist infection more successfully than others [16,17]. Recently, a GWA study from Ghana and Gambia identified rs4331426 on the chromosome 18q11.2, as a susceptibility locus associating with the risk of TB [11]. Till now, this is the only GWA study relating to the susceptibility of TB, suggesting that a new non-MHC locus can be identified in an infectious disease caused by a highly polymorphic pathogen even in African populations [11]. The identified variant of SNP rs4331426 is common in the African, but is much rarer in other populations. No data of this SNP have been published yet in association with TB from other areas of the world. Considering the limitation of extensive genetic diversity and shorter LD ranges in African populations, we performed a study to validate this finding in China by searching for tag SNPs on the chromosome 18q11.2 in the 100 Kbp up and down stream of the SNP rs4331426. To our knowledge, since the publication of the GWA study by Thye et al [11], our work is the first one to explore the role of genetic polymorphisms in this region on the susceptibility to TB. Unfortunately, we observed no significant association between TB risk and selected SNPs individually. One possible explanation might be the heterogeneity of populations, which can be confirmed by the disparity of genotype frequency [18]. Another explanation was that we only detected tag SNPs on the chromosome 18q11.2 within 100 Kbp in the up and down stream of the SNP rs4331426, which could only represent a relatively narrow scope of the genetic loci. Even though none of polymorphisms we investigated were associated with TB in the single-point locus analysis, we found the haplotypes within this block might be associated with the altered risks of TB. For example, compared to individuals carrying the common haplotype A_rs8087945_A_rs12456774_, those with A _rs8087945_G _rs12456774 _or G _rs8087945_A _rs12456774 _had a significantly decreased risk. We should notice that in this study we only analyzed two haplotypes. Other haplotypes covering more SNPs might also contribute to the risk of TB.

Interestingly, chromosome 18q11.2 is a gene-desert region that is punctuated by evolutionarily conserved domains with regulatory potential [11]. Neither rs8087945 nor rs12456774 is located inside any gene or in the regulatory sequence. The nearest genes to these SNPs are GATA6, CTAGE1, RBBP8 and CABLES1, as well as a number of as yet unannotated open reading frames. Additional studies are required to ascertain their functional significance and any possible counterbalancing selective pressures. In addition, it must be noted that the association found in China could be population-specific; however, it could also be a false-positive result. For this reason, it is important that these findings should be replicated to confirm the association in other areas of China. Future work is needed to explore the nearest genes as well as a number of as yet unannotated open reading frames around this region.

## Conclusions

Susceptibility locus of rs4331426 identified in the African population could not be validated in the Chinese population. Even though none of genetic polymorphisms we investigated was associated with TB in the single-point analysis, the haplotypes might contribute to the susceptibility to TB in the Chinese Han population. Additional studies are required to ascertain the causative variant, its functional significance and any possible counterbalancing selective pressures.

## Competing interests

The authors declare that they have no competing interests.

## Authors' contributions

JW, ZX, HP, ST, and HS implemented the field study. JW and YD performed laboratory tests. YD and JW participated in the statistical analysis and drafted the manuscript. All authors read and approved the final manuscript.

## Pre-publication history

The pre-publication history for this paper can be accessed here:

http://www.biomedcentral.com/1471-2334/11/282/prepub

## Supplementary Material

Additional file 1 **Side by side r^2^/D' plot for selected tag SNPs**. Generated by Haploview software. The 5' and 3' ends of the six SNPs are indicated. D' values are shown on the squares. The colors of the squares represent r^2 ^values, with dark being r^2 ^= 1, and white being r^2 ^= 0. Click here for file
