# Supplementary figures and images for: Fine mapping of genetic polymorphisms of pulmonary tuberculosis within chromosome 18q11.2 in the Chinese population: a case-control study
Source: BMC Infect Dis. 2011 Oct 22;11:282. doi: 10.1186/1471-2334-11-282 (PMC3248069; doi:10.1186/1471-2334-11-282)

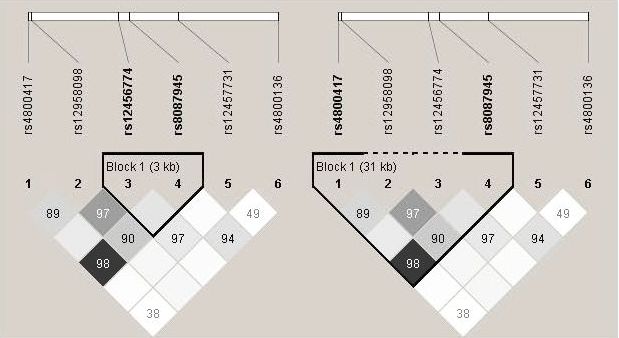

Supplement: Additional file 1 — Side by side r2/D' plot for selected tag SNPs. Generated by Haploview software. The 5' and 3' ends of the six SNPs are indicated. D' values are shown on the squares. The colors of the squares represent r2 values, with dark being r2 = 1, and white being r2 = 0. [file 1471-2334-11-282-S1.JPEG]
